# Response to ‘Values of Integrated Care: A Systematic Review’ by Nick Zonneveld et al

**DOI:** 10.5334/ijic.4723

**Published:** 2019-07-09

**Authors:** Axel Kaehne

**Affiliations:** 1 Edge Hill University, UK

4th March 2019

Dear IJIC editors,

I am writing in response to the paper published recently in your journal ‘Values of Integrated Care: A Systematic Review’ by Nick Zonneveld et al. [1].

The paper makes an important contribution to the debate around the role of values and standards of integrated care solutions in health care provision but also raises salient issues that, I believe, require further discussion and clarification.

I feel that there are two matters in particular that may benefit from elaboration by the authors. The first relates to the value (no pun intended) of conducting a systematic review of published evidence on values in integrated care research and the nature of evidence. The second is the role of values in social activities such as integrated care programmes in organisational contexts. Both matters have practical and philosophical (strictly speaking: epistemological) dimensions and, depending on how we answer the questions raised, we may be able to say more about the worth of ‘value research’ in integrated care.

First, some concerns about conducting a systematic review of values in publications. There are, of course, studies that analyse the notion of values in research and practice [2,3,4,5]. These analyses aim to exhibit the deeper political, socio-economic or epistemic structures that steer us in our studies or practical implementations of policy. Their working hypothesis is that the surface meanings of our texts (in practice and academic writing) mask rather than disclose reality. Their techniques are usually of the analytical-discursive or hermeneutic variety, carefully locating the meaning of words in a given social discourse, gauging their utility within the wider world of social conduct and examining the beneficiaries of that utility.

The need for a critical perspective is simple. Human beings profess to do a lot of things when they try to justify their actions. It is incumbent on us as researchers to critically assess these justificatory strategies.

Zonneveld et al. appear to employ insufficient criticality. Their analysis begins and ends with the surface terminology used by the studies they included in their systematic review. Unsurprisingly, the values they find underpin integrated care are the ones we all profess to pursue: being collaborative, being transparent, being empowering of others and so forth. It appears to me that Zonneveld et al. have aggregated a battery of motivational terms through which policy makers defend integrated care programmes. My hunch is that the policy language tail is wagging the proverbial practice dog here.

This leads me to the second matter of concern. Do values actually guide behaviours? The authors equate values with principles and this may raise some eyebrows amongst philosophers. However, the more important point appears to be: *how* exactly do values inform behaviours?

This is where organisational theory comes into the picture. On the individual level, values may or may not be a determinant for actions. The best we can say is that they *under*determine human behavior [6]. So, attributing any guiding force to them appears overly optimistic.

Organisations are social systems with rules, forms of stratification, control and, in health care, distinct standardised practices aligned with professional groups. Where do values feature in this and how? The fact is that we know very little about what happens when integrated care programmes collide with complex social systems such as health care organisations. In other words, we conveniently *over*estimate the behaviour defining capacity of values whilst *under*estimating the messiness of human conduct in organisational contexts.

I hope the dilemma of studies like Zonneveld et al. for advancing integrated care research has become clear. Our task is to think through the epistemological consequences of doing integrated care research at the intersection of individual and organisational conduct. Systematic reviews of value terminology in publications may be ill-suited to this endeavour.
